# Synthesis and structural characterization of Ca_12_Ga_14_O_33_

**DOI:** 10.1038/s41598-020-73311-w

**Published:** 2020-10-01

**Authors:** Sabrina E. A. McCoy, John R. Salasin, S. Michelle Everett, Claudia J. Rawn

**Affiliations:** 1 Department of Materials Science and Engineering, University of Tennessee, Knoxville, USA; 2 BWX Technologies, Lynchburg, VA USA; 3 Neutron Scattering Division, Oak Ridge National Laboratory, Oak Ridge, TN USA; 4 Center for Materials Processing, University of Tennessee, Knoxville, USA

**Keywords:** Structure of solids and liquids, Materials for energy and catalysis, Materials science

## Abstract

Ca_12_Ga_14_O_33_ was successfully synthesized using a wet chemistry technique to promote the homogenous mixing of the Ca and Ga cations. Rietveld refinements on X-ray and neutron powder diffraction data confirm that the compound is isostructural to Ca_12_Al_14_O_33,_ however, with a significantly larger lattice parameter allowing for the cages that result from the framework arrangement to expand. In naturally occurring Ca_12_Al_14_O_33_, the mineral mayenite, these cages are occupied by O^2−^ anions, however, experimental studies exchanging the O^2−^ anions with other anions has led to a host of applications, depending on the caged anion. The functional nature of the structure, where framework distortions coupled with cage occupants, are correlated to electronic band structure and modifications to the framework could lead to interesting physical properties. The phase evolution was tracked using thermogravimetric analysis and high temperature X-ray diffraction and showed a lower formation temperature for the Ca_12_Ga_14_O_33_ analogue compared to Ca_12_Al_14_O_33_ synthesized using the same wet chemistry technique. Analyzing both X-ray and neutron powder diffraction using the Rietveld method with two different starting models results in one structural model, with one Ca position and the caged O on a 24*d* special position, being preferred.

## Introduction

The atomic structure of Ca_12_Al_14_O_33_, also known as C12A7, consists of a positively charged cage-like framework where occluded anions occupy a fraction of the cages to balance the positively charged framework. The diffusion of anions from cage to cage allows the material high ion mobility and the potential for ion storage^1^. This functionality leads to applications for Ca_12_Al_14_O_33_ as a CO_2_ absorber^2^, a catalyst^3^, and as an inorganic electride^4^. The highly functional nature of the material raises questions about isostructural compounds and their potential properties. Framework distortion and cage occupancy are correlated to electronic band structure changes responsible for electrical insulator to conductor transition. Further modifications to the framework through alteration of cations and charge balancing anions in the structure should lead to changes in physical properties; the alteration of material properties with atomic substitutions has been experimentally and theoretically explored and are summarized in^5^. The ionic radius of Ga^3+^ in fourfold coordination with O is 47 pm compared to the 39 pm radius of Al^3+^^6^ suggesting that this substitution could result in a cage with a larger diameter. Partial site replacement of tri-valent Al cations has been performed with similar tri-valent Ga cations. However, results led to the conclusion that the single phase (Ca_12_Al_14-x_Ga_x_O_32_)O formed when x = 1 and when x = 2 both Ca_5_Ga_6_O_14_ and Ca_3_Al_2_O_6_ were observed in addition to Ca_12_Al_12_Ga_2_O_33_^7^. When Ca_12_Al_13_GaO_33_ was subjected to reducing conditions at 1350 °C for 6 h, buried in graphite powder, the compound decomposed to Ca_3_Al_2_O_6_ and Ca_12_Al_14−x_Ga_x_O_33_^7^. The cause of decomposition is not known, but Ca_12_Al_14_O_33_ structure in general demonstrates complicated thermodynamic instability/stability trends under dry reducing conditions above 1100 °C^8, 9^. The influence of Ga on the cage structure and localized electron behavior could alter the physical properties including the mobility of occluded species, either atomic, molecular, or electrons, and the electrical conductivity.

The existence of Ca_12_Ga_14_O_33_ has been theorized due to the similarity of the CaO–Al_2_O_3_ and CaO–Ga_2_O_3_ binary phase diagrams and existence of isostructural compounds from the two systems including Ca_5_Ga_6_O_14_ and CaGa_4_O_7_. Many versions of the CaO–Al_2_O_3_ binary phase diagram exist due to the importance of CaO–Al_2_O_3_ compounds to the cement industry. Using the Phase Diagrams for Ceramist online database 24 CaO–Al_2_O_3_ phase diagrams result with the earliest dating back to a 1955 dissertation^10^ followed by a 1956 version in the 2nd Edition of *The Chemistry of Cement and Concrete*^11^. The latter shows the Ca_12_Al_14_O_33_ compound and although some of the details have been refined the overall phase diagram remains generally unchanged. In 2001 Jerebstov and Mikhailov^12^ published the CaO-Al_2_O_3_ diagram developed under anhydrous conditions and without moisture was not able to synthesis the Ca_12_Al_14_O_33_ compound supporting an earlier observation by Nurse et al.^13^. In contrast when using the Phase Diagrams for Ceramist online database three CaO–Ga_2_O_3_ phase diagrams result. All three phase diagrams^14–16^ show the compounds Ca_3_Ga_2_O_6_, CaGa_2_O_4_, and CaGa_4_O_7_ and the phase diagrams by Young^15^ and Kovba et al.^16^ show Ca_3_Ga_4_O_9_ as a fourth intermediate compound. Conducting a database search of CaO–Ga_2_O_3_ compounds using the Inorganic Crystal Structure Database (ICSD)^17^ two other intermediate compounds, Ca_2_Ga_2_O_5_^18^ and Ca_5_Ga_6_O_14_^19–21^ have been reported, the former being synthesized with high-pressure. Londar^22^ grew Ca_5_Ga_6_O_14_ crystals using the Czochralski method and starting with a 12CaO:7Ga_2_O_3_ mixture. Tolkacheva et al.^23^ synthesized Ca_5_Ga_6_O_14_ using solid state synthesis techniques and characterized the compound using X-ray diffraction, infrared and Raman spectroscopy, differential scanning calorimetry, thermogravimetric analysis, density measurements, and dilatometry. The latter was studied in both dry and moist atmospheres and after the sample was heated under moist conditions X-ray diffraction revealed CaGa_2_O_4_, Ga_2_O_3_, and CaCO_3_, thought to have transformed from CaO during cooling in an environment where CO_2_ was present.

It has previously been identified that the Ca_5_Al_6_O_14_ phase forms through decomposition kinetics of Ca_12_Al_14_O_33_, indicating that Ca_12_Ga_14_O_33_ may be stable at low temperatures^8, 24^. Similar to Ca_12_Al_14_O_33_ it is expected the thermodynamic stability of the Ca_12_Ga_14_O_33_ (ρ = 3.54 g/cm^3^) will be dependent on availability of anions that are able to stabilize cage structure over the layered and more dense CaGa_4_O_7_ (ρ = 4.46 g/cm^3^), Ca_3_Ga_4_O_9_ (ρ = 4.21 g/cm^3^), and Ca_5_Ga_6_O_14_ (ρ = 4.12 g/cm^3^) phases.

Here we report on the successful synthesis of Ca_12_Ga_14_O_33_ through a solution-based route, provide a crystal structure characterization from X-ray and neutron powder diffraction data, evaluate the phase formation through thermogravimetric analysis (TGA) and high temperature X-ray diffraction (HTXRD) studies, and compare and contrast the Ca_12_Al_14_O_33_ and Ca_12_Ga_14_O_33_ compounds. Solution synthesis allows for homogenous mixing of the metal atoms, shortening diffusion pathways and allowing for kinetically favorable, but thermodynamically unfavorable, phases to form.

## Experimental and methods

The polymer assisted sol–gel synthesis method was employed utilizing poly vinyl alcohol (PVA)^25^. The 88% hydrolyzed PVA, with molecular weight between 20,000 and 30,000 g/mol, was dissolved in deionized water and allowed to stir for 1 h and Ca(NO_3_)_2_·4H_2_O (ACS Grade, Fisher Chemical) and GaCl_3_ (99.99 +%, Acros Organics) were dissolved separately into deionized water. The stoichiometry of the solutions was characterized through gravimetric titration. For synthesis, stoichiometric amounts of the Ca(NO_3_)_2_ and GaCl_3_ solutions were added to the PVA solution and allowed to stir for 1 h prior to heating. A 4:1 ratio of the number of metal cations to PVA monomer units was used as previously identified as the ideal amount for Ca_12_Al_14_O_33_^8^. The solution was heated on a 300 °C hotplate until most of solvent evaporated and the solution became viscous. The viscous liquid was placed in a 120 °C drying oven for 12 h and dried to a light foam; foaming occurs due to the evaporation of nitrate and chloride species. The foam was ground to a fine powder and divided for the various characterization studies.

Thermogravimetric Analysis (TGA) was performed on the dried non calcined polymer powder using a TA Instruments Q50 TGA. The sample was heated at a constant rate of 10 °C/min from 25 to 650 °C. Mass loss was recorded as a function of temperature.

A fraction of the powder was then calcined to 600 °C, based on the TGA data, to decompose the organics to allow for clean processing in the XRD environmental chamber, and immediately quenched. The resulting calcined powder was pressed into a 13 mm pellet. The remaining organic/inorganic PVA powder was retained for further thermal processing. Both room temperature and HTXRD data were collected using a Malvern PANalytical Empyrean diffractometer with a Cu radiation source operating with an accelerating voltage of 45 kV and current of 40 mA. A PIXcel^3D^ area detector with 255 active channels with ~ 3°2*θ* of coverage was used for rapid non-ambient data collection. For non-ambient data collection the sample was heated at 5 °C/min up to 800 °C, then the rate was slowed to 1.5 °C/min as the sample was heated to 1000 °C. Data were collected in the range from 25 to 36.5°2*θ,* this region contains major peaks related to the Ca_12_Ga_14_O_33_ phase and possible secondary phases including Ga_2_O_3_, Ca_5_Ga_6_O_14_, CaGa_2_O_4_, CaGa_4_O_7_, and Ca_3_Ga_4_O, and CaO. Using a 0.0131° step size and 13.77 s counting time each scan was approximately 1.5 min allowing for phase transitions to be detected while they occur. A longer data collection range, from 15–80°2*θ*, was used at 1000 °C to determine the full diffraction pattern and evaluate the evolved phases. The non-calcined powder was pressed into a pellet and was fired at 800 °C for 1 h. Subsequently ambient temperature data were collected from 15–120°2*θ* in high spatial resolution mode to verify the structure of the material and allow for refinement on the structural details including the lattice parameter, atomic positions, and atomic displacement parameters.

Time-of-flight powder neutron diffraction data were collected on the Nanoscale Ordered Materials Diffractometer (NOMAD) beamline^26^ of the Spallation Neutron Source (SNS) at Oak Ridge National Laboratory (ORNL). About 1.5 g of near single-phase sample was contained in a 6 mm V sample canister. The partially calcined powder was fired at 800 °C for 1 h prior to loading the sample canister. NOMAD’s detectors were calibrated using diamond, and silicon was used to generate the starting instrumental parameters. S(Q) was produced through normalizing the sample scattering via a solid vanadium rod and subtracting the background collected for an empty 6 mm V sample canister. Four individual data sets were collected and merged together for the data analysis.

HighScore Plus software package^27^ interfaced with the ICDD PDF4 + database^28^ was used for phase identification and the GSAS II software package^29^ was used for the analyzing both the XRD and neutron diffraction data using the Rietveld method. For the data collected the lowest possible R obtainable for the data or wR_min_, based in part on the number of data points and calculated in GSAS II, was 10.39% for the XRD data and 0.43% for the NPD data.

## Results and discussion

### TGA characterization

**Figure 1:**
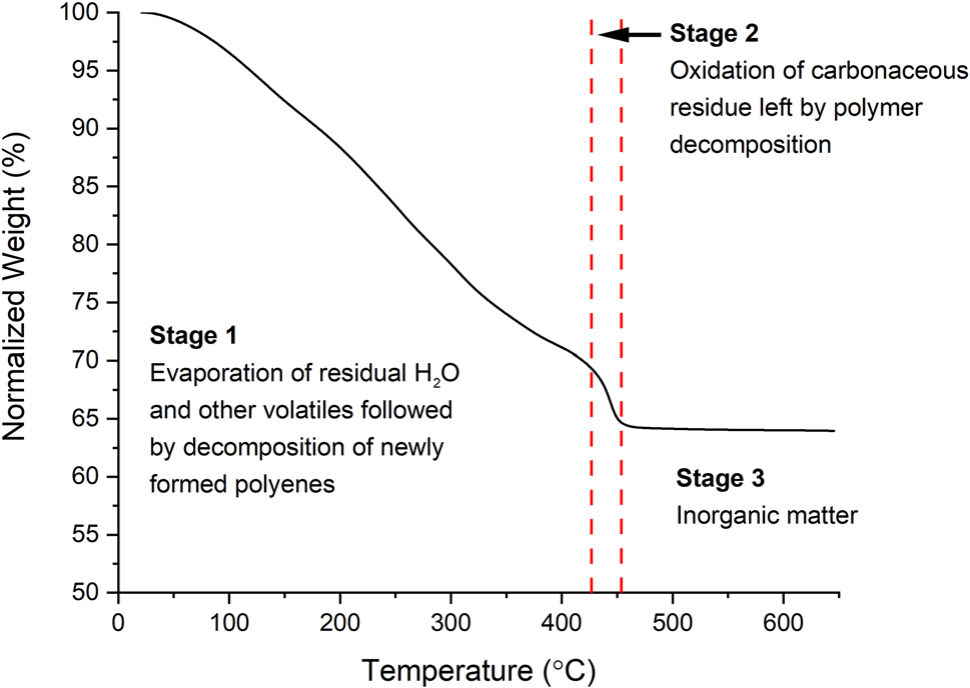
TGA showing the decomposition of the organics to inorganic matter from the unfired material.

Thermogravimetric analysis of the Ca_12_Ga_14_O_33_ PVA powder, shown in Fig. 1, displays three decomposition stages during the heating. The first stage, from 25 °C to approximately 425 °C, shows the evaporation of residual H_2_O and other volatiles remaining in the sample. When PVA is heated, polymer condensation reactions occur releasing structurally trapped water and causing polyenes to form and additional weight loss is due to the decomposition of the newly formed polyenes^25^. In this region the decomposition follows a relatively constant slope, indicating that decomposition is uniform as the temperature increases. This is ideal for preventing segregation of species within the sample during calcination. The sharp drop in mass above 425 °C is likely caused by the oxidation of carbonaceous residue left by polymer decomposition^25^. The sample experiences no further weight loss above 450 °C indicating that the remaining components in the sample are inorganic. The TGA results suggest that between 500 and 600 °C is the optimal temperature for calcination to ensure that all organics decompose. The sample experienced a total weight loss of 35% making this a high yield solution-based synthesis method.

### XRD characterization

**Figure 2:**
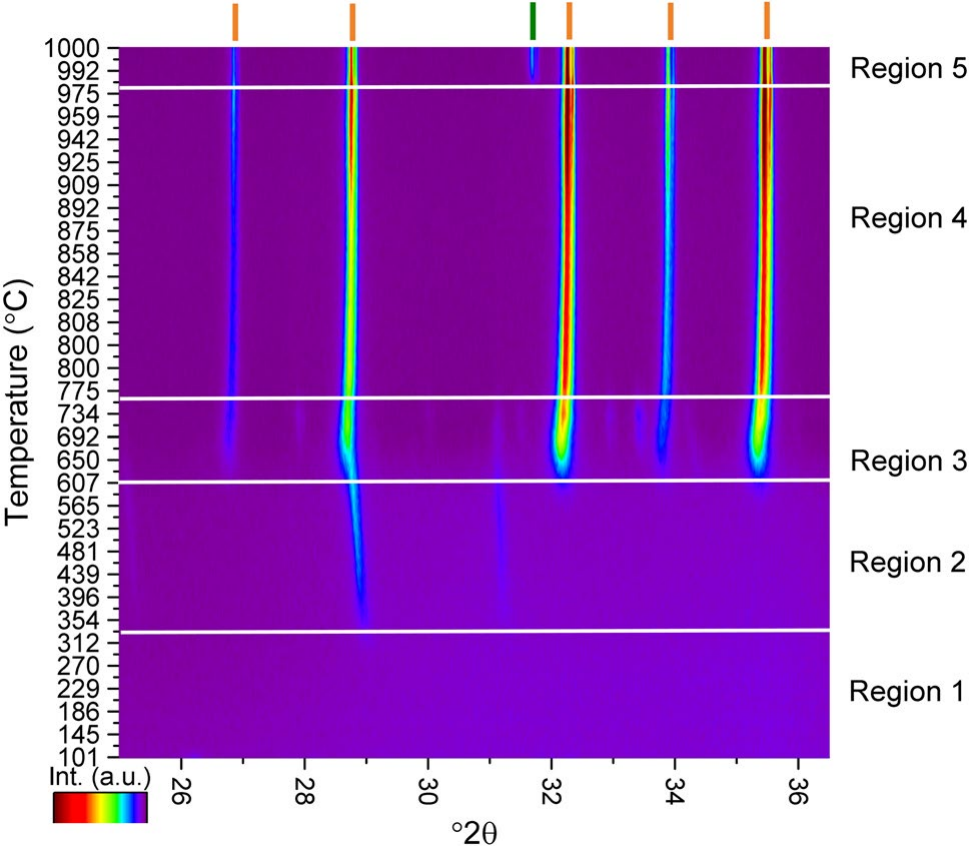
Intensity map of the HTXRD data from the pellet calcined at 600 °C. The orange dashes indicate the Ca_12_Ga_14_O_33_ phase and the green dashes mark the CaO phase. Heatmap generated using the HighScore Plus software package^27^, dash marks for indicating the CaO and Ca_12_Ga_14_O_33_ phases determined from the ICSD^17^ and ICDD^28^ databases.

**Figure 3:**
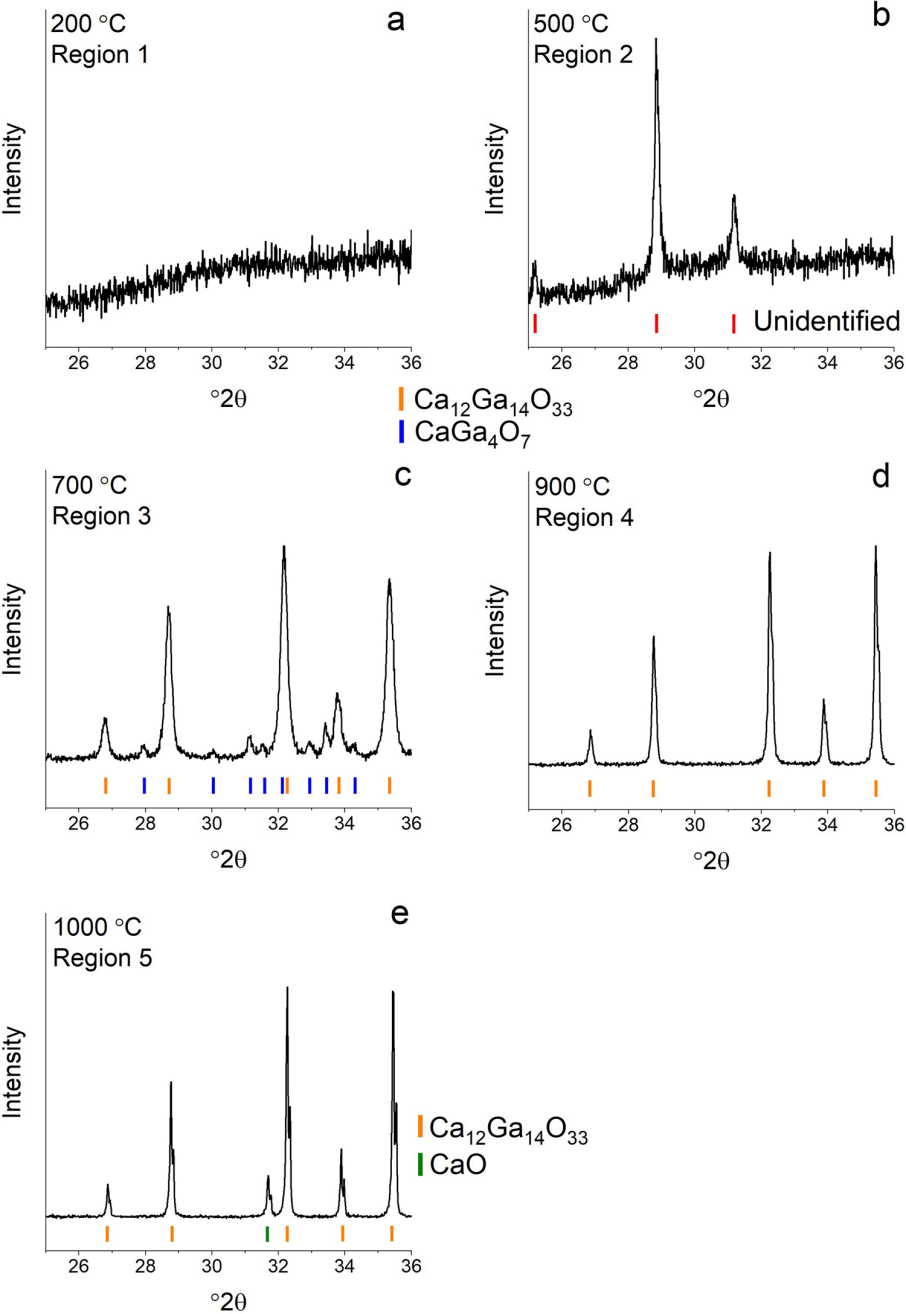
Representative X-ray powder diffraction data from selected temperature regions identified from the HTXRD results. Data collected at (**a**) 200 °C, (**b**) 500 °C, (**c**) 700 °C, (**d**) 900 °C, and (**e**) 1000 °C. Dash marks for indicating the CaO, CaGa_4_O_7_, and Ca_12_Ga_14_O_33_ phases determined from the ICSD^17^ and ICDD^28^ databases.

**Figure 4:**
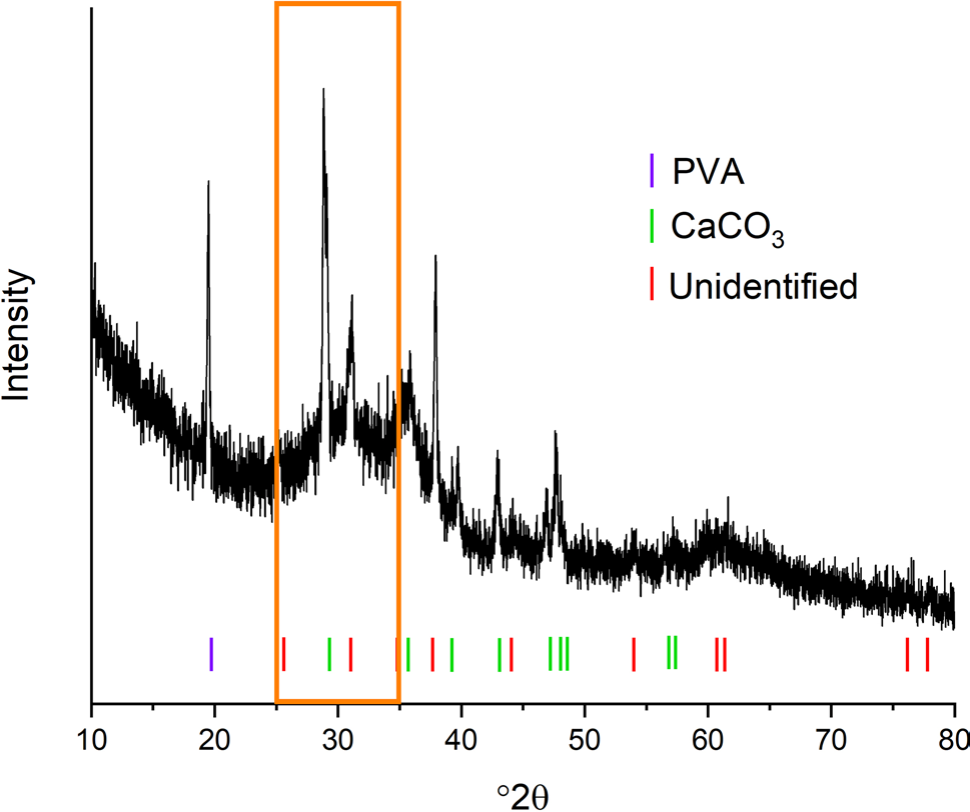
Larger data collection range collected at 500 °C. The region in the box corresponds to the region collected during the fast data collection (Fig. 3b). Data collected on PVA and peak markers for CaCO_3_ are included for comparison. Dash marks for indicating the PVA and CaCO_3_, phases determined from the ICSD^17^ and ICDD^28^ databases.

In-situ high temperature X-ray diffraction was used to observe phase transformations during heating in the data range from 25 to 36.5°2*θ*. The intensity heat map of the scans is shown in Fig. 2 and has five distinct phase regions. Representative patterns from each temperature region are shown in Fig. 3. Data collected on the 600 °C pre-calcined pellet shows that at temperatures below approximately 300 °C the sample is amorphous (Fig. 3a). After calcination most of the organics have been removed and a structureless mix of metal and oxygen atoms remains, with no diffraction peaks present in the data range. When heated above approximately 300 °C, two peaks appear at 28.9 and 31.2°2*θ *(Fig. 3b). In an attempt to identify the phase(s) that form between 300 and 650 °C data were collected in a larger °2*θ* range, between 10–80°2*θ*, and is shown in Fig. 4, revealing additional peaks associated with the phase(s). Despite attempts using the HighScore Plus software and the ICDD PDF4 + database, with and without chemical constraints, the peaks remain unidentified and could be representing either a single phase or a multiphase mixture. The sample had previously been heated to 600 °C during calcination to remove the organics, so this phase transformation is likely an atmospheric response of the amorphous Ca, Ga, and O species. At approximately 650 °C the unknown phase(s) disappear(s) and a multiphase mixture forms with peaks suggesting a compound isostructural to the Ca_12_Al_14_O_33_, with Ga substituted on the Al sites, along with CaGa_4_O_7_ (ICSD 10351) as a minor secondary phase (Fig. 3c). The peaks belonging to the Ca_12_Ga_14_O_33_ compound get sharper and more intense as the temperature increases, consistent with phase and crystallite growth. CaGa_4_O_7_ is present between 650 and 750 °C disappearing above 750 °C. The presence of this Ga rich phase could indicate a non-equilibrium assemblage that forms due to the high heating rate or potentially during the solution-based synthesis where nonstoichiometric areas of the precursor form but get quickly removed when the diffusion increases with higher temperature. Above 750 °C (Fig. 3d) single phase Ca_12_Ga_14_O_33_ is present until a new peak at approximately 31.5°2*θ* appears at 975 °C (Fig. 3e). To identify the additional phase that is represented by the new peak, data were collection on a larger °2*θ* range (10–80°2*θ*) at 1000 °C. It was confirmed that the peak belongs to the CaO and using the Rietveld technique results in 19.8(3) wt% of CaO with all peaks in the pattern accounted for by either Ca_12_Ga_14_O_33_ or CaO.

A pellet of the amorphous sol–gel reactants was fired at 800 °C to form Ca_12_Ga_14_O_33_ and high spatial resolution X-ray diffraction data were collected on the powdered sample at room temperature to verify the phase purity and characterize the crystal structure. To verify the crystal structure Rietveld refinements using two different models, based on the Ca_12_Al_14_O_33_ structure, were attempted. The two slightly different atomic structures were reported by Bartl and Scheller^30^, determined from single crystal X-ray diffraction data, and Boysen et al.^31^ (ICSD 241000) determined from neutron powder diffraction data. Both structures crystallized in the cubic crystal system, space group $I\overline{4}3d$ (space group number 220) and with a unit cell edge close to 12 Å. The structure by Boysen et al*.*^31^ differed from the structure by Bartl and Scheller^30^ by having two unique partially occupied Ca positions on 24*d* sites as opposed to one fully occupied Ca 24*d* site. Another difference between the two reported structures is that Bartl and Scheller^30^ placed the caged O on a 24*d* site (x, 0, ¼, specifically 0.337, 0, ¼) with a corresponding site occupancy of 0.083 for filling approximately two of the 12 cages/unit cell. Boysen et al.^31^ working with neutron powder diffraction data, which is more sensitive to the O atoms (the bound coherent neutron scattering lengths for Ca, Al, and O are 4.70, 3.449, and 5.803 fm, respectively^32^) moved the caged O from 0.337 to 0.375 or the 12*a* site ($\frac{3}{8}$, 0, ¼) with a correspondingly higher site occupancy for filling approximately two of the 12 cages/unit cell. Additional studies have been carried out using synchrotron radiation to understand the details of the cage distortions and cage occupancy^33, 34^. The intent of the characterization studies presented here is not to provide crystallographic details but to confirm the isostructural nature of Ca_12_Ga_14_O_33_ to Ca_12_Al_14_O_33_ and the model that fits best.

**Figure 5:**
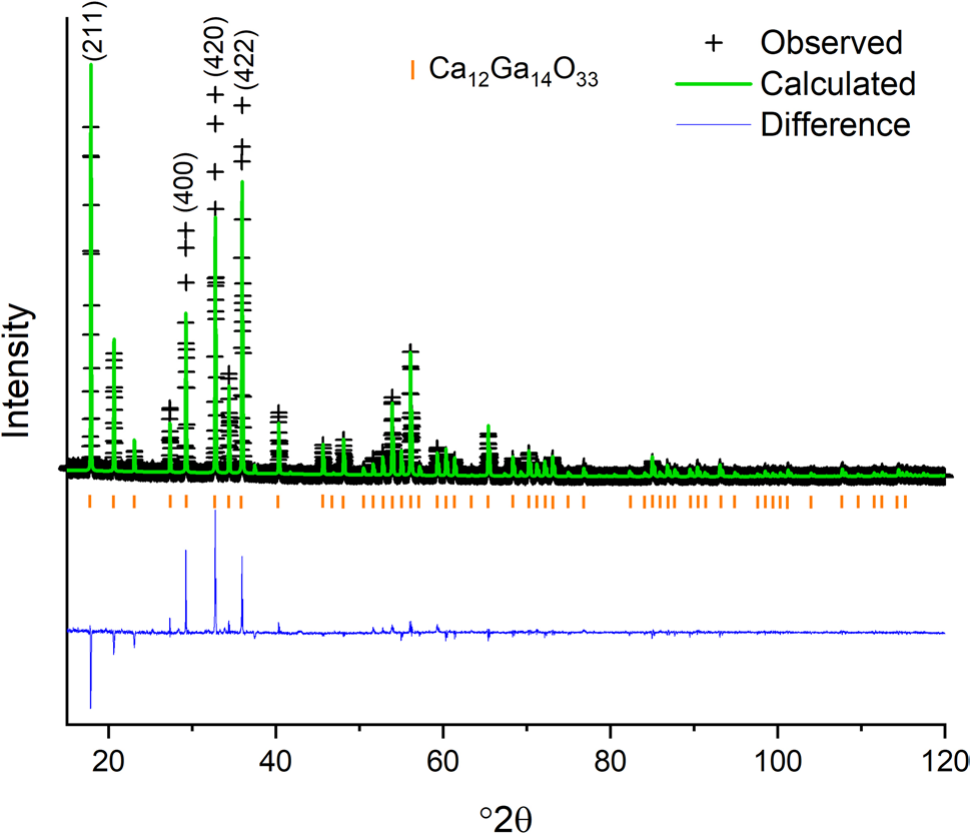
Room temperature XRD data (black crosses), calculated XRD pattern based on the atomic structure with two partially occupied Ca positions and the caged oxygen on the 12*a* site (green line) and difference pattern (blue line). Graphic generated using the GSAS II software package^29^, dash marks for indicating the Ca_12_Ga_14_O_33_ phase determined from the ICSD^17^ and ICDD^28^ databases.

**Figure 6:**
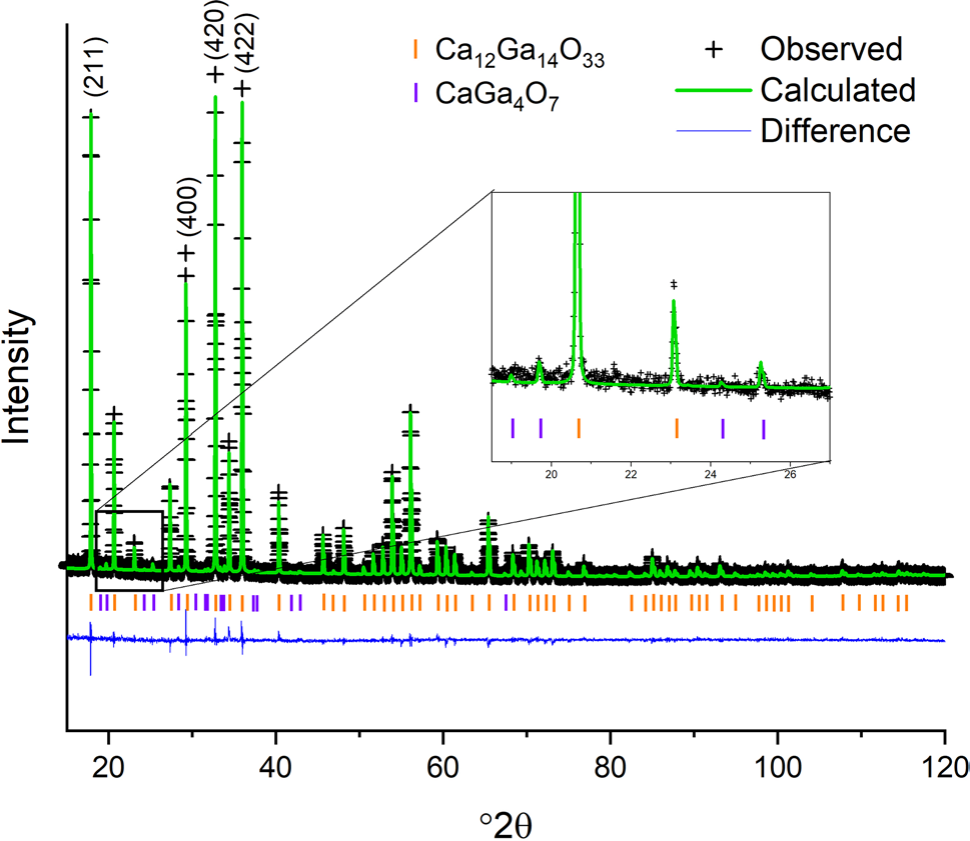
Room temperature XRD data (black crosses), calculated XRD pattern based on the atomic structure with one unique Ca position and the caged oxygen on the 24*d* site (green line) and difference pattern (blue line). The inset shows a smaller range that contains peaks belonging to CaGa_4_O_7_. Graphic generated using the GSAS II software package^29^, dash marks for indicating the Ca_12_Ga_14_O_33_ and CaGa_4_O_7_ phases determined from the ICSD^17^ and ICDD^28^ databases.

When using the structure with the two unique partially occupied Ca sites and caged O on the 12*a* site^31^ and changing the Al atoms to Ga atoms, it was impossible to refine on the atomic displacement parameter (adp) for the O atom in the cage center without the isotropic atomic displacement parameter (U_iso_) going negative. If the adps for all three oxygens in the structure were constrained together, a positive U_iso_ was obtained but it was impossible to refine on the site occupancy factor of the caged oxygen and obtain a physically reasonable result. When the site occupancy factor was fixed at $\frac {1} {6}$ and the U_iso_ of the three oxygens constrained together, plausible results were obtained, however, there were intensity mismatches, shown in Fig. 5, for several of the peaks including the (211), (400), (420), and (422). The resulting agreement factor for the refinement is wR = 18.54% for a goodness of fit (GOF) (wR/wR_min_) of 1.79. When using the structure with only one Ca position and the caged O on a 24*d* site^30^ and changing the Al atoms to Ga atoms it was possible to refine on the adps of the oxygen atoms separately and the site occupancy factor of the caged oxygen. The intensity differences, between the calculated XRD pattern and the observed data, for the reflections discussed above were smaller, as shown in Fig. 6, and a better overall structural model is supported by the resulting smaller wR = 14.94% and the GOF = 1.44. Upon close examination there were a few additional weak peaks but these were accounted for by adding a small amount of CaGa_4_O_7_^35^ as a secondary phase, resulting in a wR = 13.75% and GOF = 1.33 with 1.9(2) wt% CaGa_4_O_7_. Experimental and refinement details, crystal data, and refined fractional coordinates, site occupancy factors, and isotropic thermal parameters are given in Tables 1, 2, and 3, respectively. The above suggests the better structural model for Ca_12_Ga_14_O_33_ is the model with only one Ca position and the caged O in the 24*d* site^30^. However, given the low occupancy of any atomic or molecular species occluded in the cage, coupled with the occluded species having fewer electrons than the framework elements, Ca and Ga, the best structural model will depend on the elusive occluded atomic or molecular species.

**Table 1:** Experimental information, refinement parameters, and results.

	XRD		NPD	
**Model**	2 Ca	1 Ca	2 Ca	1 Ca
Caged O site	12*a*	24*d*	12*a*	24*d*
Goodness of fit, GOF	1.79	1.33	16.59	16.61
wR (%)	18.54	13.75	4.83	4.84
Instrument type	Bragg–Brentano		Debye–Scherrer	
Data range	15–120°2*θ*		0.4–5 *d* (Å)	
**Variables**	17	28	59	57
Background	3	3	24	24
Lattice parameter^a^	12.2992(3)	12.2993(3)	12.316(3)	12.316(3)
Sample parameters				
Histogram Scale	1.824(9)	2.093(9)	1/bank	1/bank
Sample displacement (μm)	− 472.9(7)	− 472.8(6)		
Instrument parameters	1	3	14	14
Atomic positions	7	7	7	7
Adps (U_iso_)	4	6	6	6
Site occupancy factors (caged O)	0	1	1	1
Second phase (CaGa_4_O_7_) wt%	–	1.92(9)	–	–
Lattice parameters for CaGa4O7, *a, b, c,* and β	–	4	–	–

^a^Lattice parameter estimated standard deviations are reported as 3σ.

**Table 2:** Crystal data for Ca_12_Ga_14_O_33_.

Color	White
Crystal system	Cubic
Space group	$I\overline{4}3d$(220)
*a* (Å)	12.2993(3)
Volume (Å^3^)	1860.5(2)
Z	2
Formula/refined formula	Ca_12_Ga_14_O_33_/Ca_12_Ga_14_O_39.5_
Formula weight/refined (g/mol)	1985.025/2081.019
Calculated density/refined (g/cm^3^)	3.54/3.71

**Table 3:** Refined fractional coordinates, site occupancy factors (sof) and isotropic thermal parameters (U_iso_).

Atom	Mult/Wy	x	y	z	sof	U_iso_
**X-ray powder diffraction data**						
Ca	24*d*	0	$1/4$	0.1402(3)	1	0.0234(13)
Ga	16*c*	0.01796(12)	0.01796(12)	0.01796(12)	1	0.0231(8)
Ga	12*b*	$- 1/8$	0	$1/4$	1	0.0239(11)
O	48*e*	0.1542(5)	− 0.0356(6)	0.0661(6)	1	0.029(3)
O	16*c*	− 0.0611(8)	− 0.0611(8)	− 0.0611(8)	1	0.038(6)
O	24*d*	0.4(7)	0	$1/4$	0.625(14)	0.113(13)
**Time-of-flight neutron powder diffraction data**						
Ca	24*d*	0	$1/4$	0.1433(3)	1	0.017(1)
Ga	16*c*	0.0190(1)	0.0190(1)	0.0190(1)	1	0.0058(5)
Ga	12*b*	$- 1/8$	0	$1/4$	1	0.0055(6)
O	48*e*	0.1559(2)	− 0.0288(2)	0.0633(2)	1	0.0142(5)
O	16*c*	− 0.0654(2)	− 0.0654(2)	− 0.0654(2)	1	0.030(1)
O	24*d*	0.38(2)	0	$1/4$	0.610(9)	0.046(3)

### Neutron powder diffraction characterization

**Figure 7:**
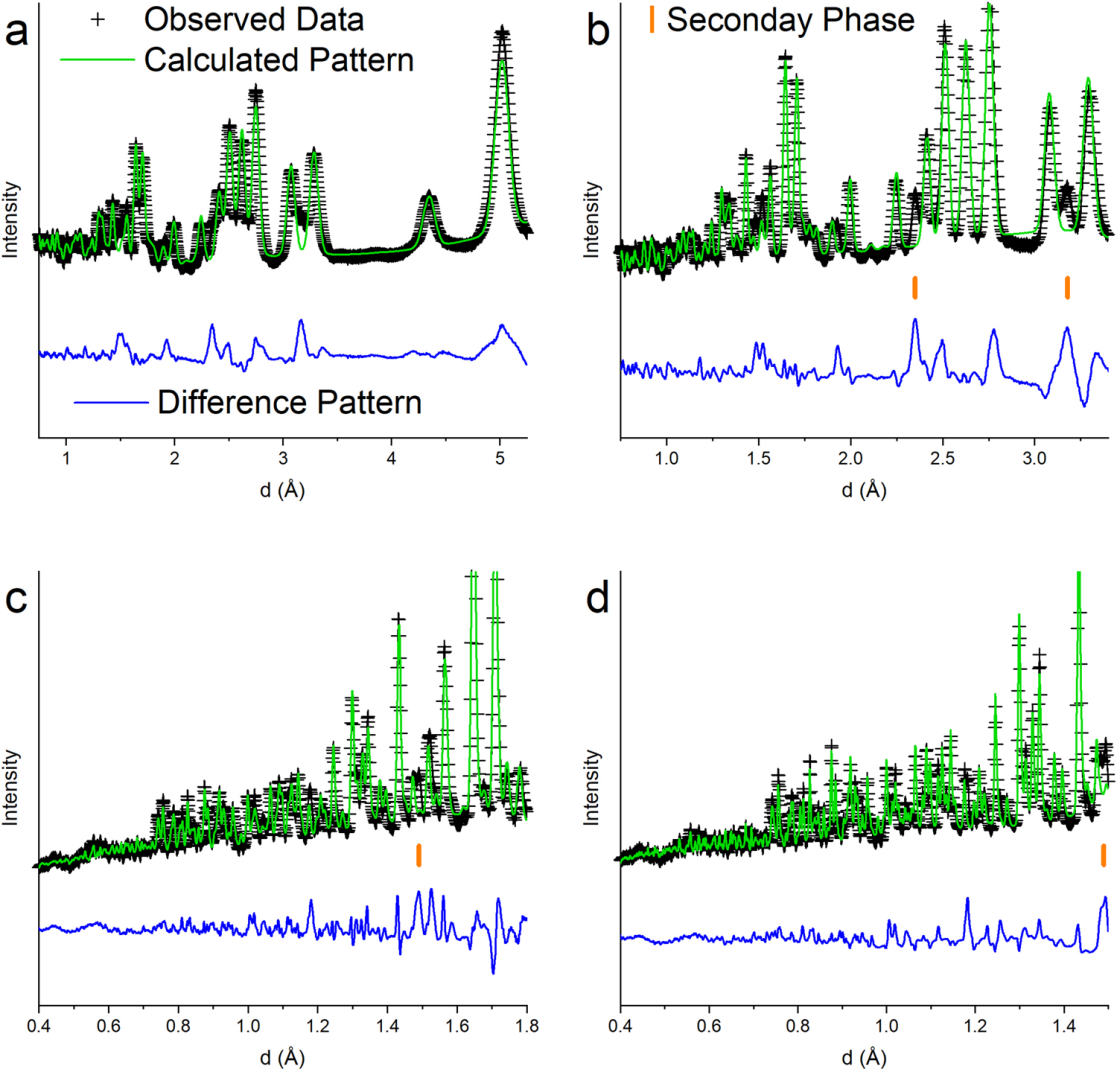
Room temperature time-of-flight neutron powder diffraction data (black crosses) from four detector banks, calculated NPD pattern based on the atomic structure with one unique Ca position and the caged oxygen on the 24*d* site (green line) and difference pattern (blue line). Graphic generated using the GSAS II software package^29^. Unidentified peaks are marked by the orange dash marks.

**Figure 8:**
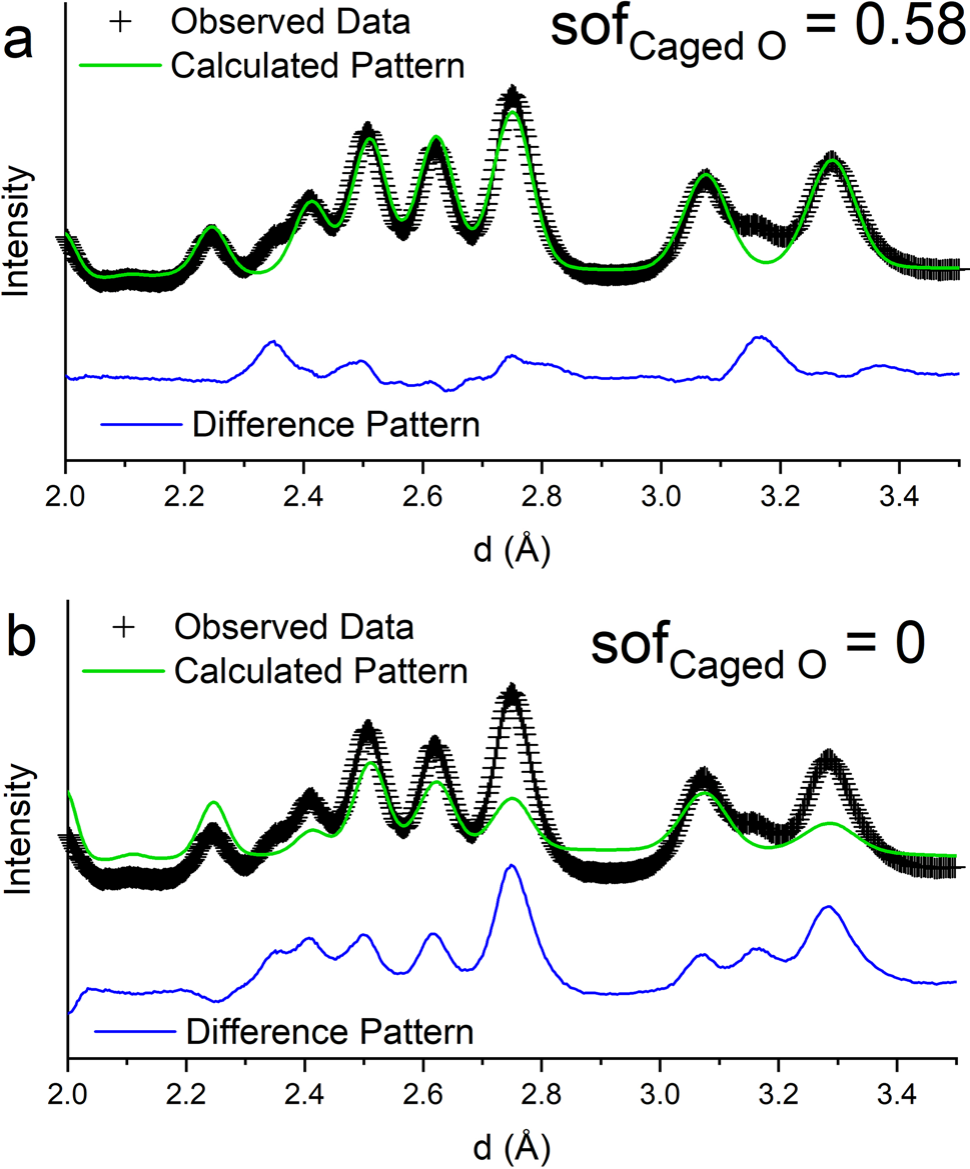
Comparison of the observed data (black crosses) to calculated time-of-flight neutron powder diffraction patterns where the occupancy of the caged O is varied (**a**) sof = 0.58 and (**b**) cage is empty (sof = 0). Graphic generated using the GSAS II software package^29^.

Four detector banks of time-of-flight neutron powder diffraction data were collected at room temperature. The data show an elevated background, approximately four times that of the background of the data collected on the Si Standard Reference Material (SRM) (NIST Si 640 E). One reason for the elevated background could be the occupational disorder of the caged O coupled with positional disorder detailed by^31, 32^. Both crystallographic models^30, 31^ discussed above were attempted to determine which structural model provided a better fit to the neutron diffraction data. The refinement agreement factors using the model with one Ca position and caged O in the 24*d* site^30^ were almost identical (wR = 4.84% and GOF = 16.61 refining on 57 variables) to those obtained using the crystallographic structural model with two unique Ca sites and the caged O on the 12*a* site^31^ (wR = 4.83% and GOF = 16.59 refining on 59 variables). However, using the model with the two unique Ca positions and the caged O on the 12*a* site when the site occupancy of the caged O was refined it resulted in a value > 1 and poor background fitting using the same function and number of coefficients. The refined lattice parameter obtained from the neutron powder diffraction data, *a* = 12.316(3) Å (lattice parameter esds reported as 3σ), is significantly larger than the lattice parameter, *a* = 12.2993(3) Å, refined based on the X-ray diffraction data. Figure 7 compares the calculated patterns, generated from the refined variables, to the observed data in each of the four detector banks. The neutron powder diffraction data show a few unexplained peaks, marked in Fig. 7, at *d* = 3.17, 2.35, and 1.49 Å [the peak at d = 1.49 Å could be the (300)], suggesting a secondary phase is present. The X-ray diffraction data show secondary phase peaks belonging to CaGa_4_O_7_ and this phase was incorporated into the Rietveld analysis and the phase fraction was refined to 1.9(2) wt%. The phase diagrams suggest either CaGa_4_O_7_, CaGa_2_O_4_ or Ca_3_Ga_4_O_9_ (depending on which phase diagram is correct) should be present in equilibrium, however, none of these compounds have reflections at or near 3.17 and 2.35 Å. Other compounds that were considered included Ca_5_Ga_6_O_14_, CaO, and CaCO_3_ (calcite) but none of these compounds matched the unexplained peaks. Calcite showed the most promise, however, the lattice parameters would need to be shifted far off of the literature values. The search was also opened up to both compounds with the calcite structure but a larger unit cell volume as well as Ca, Ga, O, and H compounds (e.g. Ca(OH)_2_, CaO_2_·8H_2_O, etc.) but no promising matches were identified. The difficulty in determining secondary phases in this study could be due to the ability to definitively determine amount of cage occupant, to charge balance $\frac {1}{6}$ of the cages are occupied by a O^2−^ anion. When using the structural model with one Ca position and the caged O on the 12*a* position^30^ the site occupancy fraction (sof) of the caged oxygen refined to 0.610(9), only slightly lower than that determined by the X-ray powder diffraction study (sof = 0.62(1)), and still high compared to sof = 0.167 that should result if one out of every six cages are occupied. Figure 8 illustrates how manually changing the site occupancy of the caged oxygen atom has a significant impact on several of the peaks in the neutron powder diffraction pattern. Future work using total scattering or pair distribution function (pdf) analysis on time-of-flight neutron powder diffraction data will reveal more information for a better understanding of the nanoscale structural features of the compound and specifically about the atom(s) present in the Ca_12_Ga_14_O_33_ cages. The results presented here are only to provide a second characterization technique that supports the synthesis of Ca_12_Ga_14_O_33_.

## Comparison of Ca_12_Ga_14_O_33_ to Ca_12_Al_14_O_33_ and conclusions

**Figure 9:**
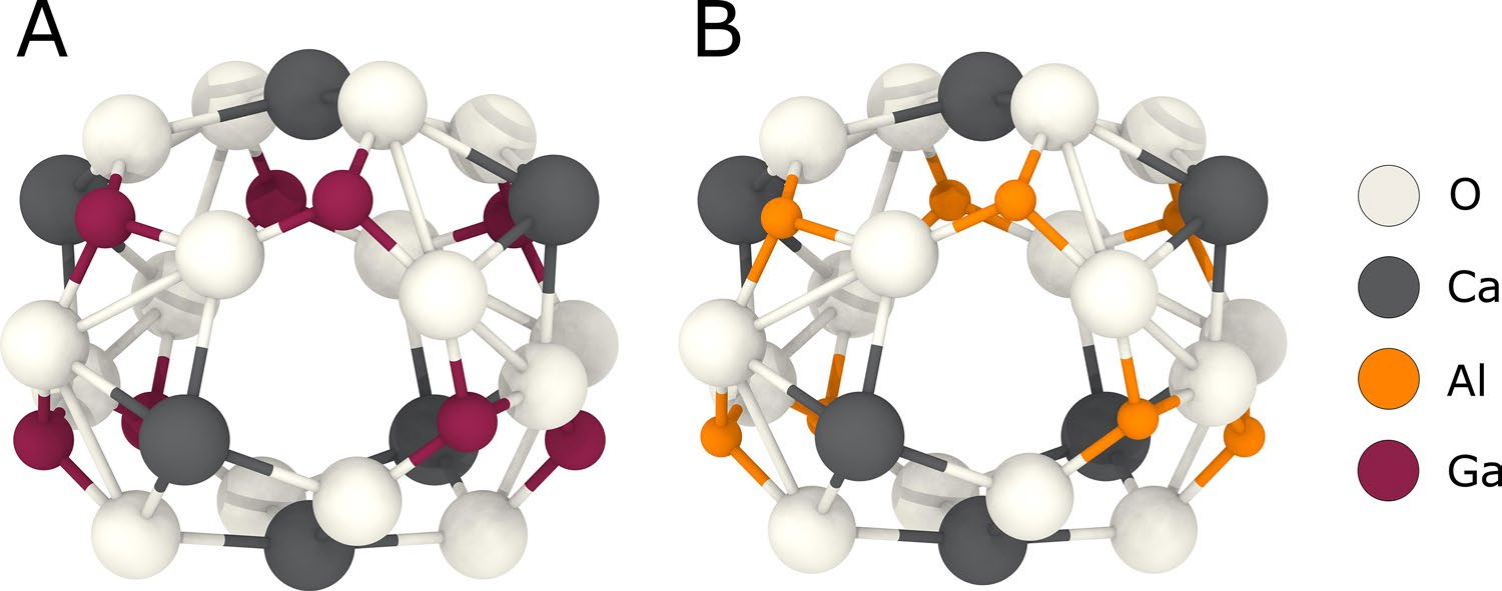
Comparison of Ca_12_Al_14_O_33_ (**A**) and Ca_12_Ga_14_O_33_ (**B**) cages. The distance between the Ca at the top and bottom of the cage, indicated by the dashed line, is 5.642 Å in the Ca_12_Al_14_O_33_ cage and 5.734 Å in the Ca_12_Ga_14_O_33_ cage. This corresponds to a 1.6% expansion in the cage diameter. Graphic generated using the OVITO software package^36^.

Ca_12_Ga_14_O_33_ was successfully synthesized using the polymer-assisted steric entrapment method^25^. X-ray and neutron diffraction data indicate that the new compound is isostructural to Ca_12_Al_14_O_33_ with a full exchanged of Al to Ga. HTXRD showed a formation temperature of 650 °C, which is significantly lower than that observed for Ca_12_Al_14_O_33_ synthesized in a similar manner^8^. The best refinement, based on both the laboratory XRD and time-of-flight neutron powder diffraction data, resulted with the model that only has one Ca position and the caged O on the 24*d* site. The refined lattice parameter for the new Ca_12_Ga_14_O_33_ compound is *a* = 12.2993(3) Å (XRD), approximately 2.6% larger than the 11.9794 Å of Ca_12_Al_14_O_33_^31^. This expansion in lattice parameter leads to a 1.6% expansion of the cages as shown in Fig. 9. The larger framework and cages potentially open the possibility of occluding larger molecular species within the cage. Future work is needed to elucidate the current cage occupants, better understand the local structure of the compound, determine potential processing conditions that converts the compound into an electride structure, and evaluate the changes in electrical properties based on cage occupants.
